# Macrophage polarization regulates bone homeostasis: a potential etiology and therapy for postmenopausal osteoporosis

**DOI:** 10.3389/fimmu.2026.1826988

**Published:** 2026-04-30

**Authors:** Quezhu Danzeng, Fanyuan Wu, Mengying Cui, Yi Shen, Yuhao Li, Jianhang Jiao, Weibo Jiang

**Affiliations:** 1 Orthopaedic Medical Center, The Second Hospital of Jilin University, Changchun, China; 2 Department of Hematology and Oncology, The Second Hospital of Jilin University, Changchun, China; 3 Department of Hepatobiliary and Pancreatic Surgery, The Second Hospital of Jilin University, Changchun, China

**Keywords:** estrogen, immunomodulatory biomaterials, macrophage, osteoblast, osteoclast, postmenopausal osteoporosis

## Abstract

Osteoporosis is a very common disease nowadays, and it is mostly seen in postmenopausal women. In postmenopausal women, with the decrease of estrogen, directly or indirectly, it makes the decrease of bone mineral density more significant, and increases the loss of bone mass, so the osteoporosis due to the decrease of estrogen is called postmenopausal osteoporosis. Macrophages play an important role in the regulation of bone metabolism, and different polarization states have different effects, promoting bone resorption when polarized to M1-type macrophages and bone formation when polarized to M2-type macrophages. In terms of bone metabolism regulation, these two polarized states are functionally opposed, yet they can transition into one another, forming a dynamic continuum; M0, M1, and M2 can all polarize one another. With the rapid development of bone immunology in recent years, this paper will first focus on macrophages, detailing their effects on osteoblasts, osteoclasts, angiogenesis, and anastomosis under different polarization states. It will then discuss estrogen, a key regulatory factor involved in both macrophage polarization and bone homeostasis, with a particular emphasis on its enhancing effects on immune cells (especially its protective role for M2 macrophages), its regulation of cytokines (directly or indirectly reducing the expression of RANK, M-CSF, IL-1, IL-6, and TNF-α, while increasing OPG expression), as well as its role in promoting angiogenesis through increased VEGF production and protecting blood vessels to exert anti-inflammatory effects. Current treatments are primarily pharmacological and faces challenges such as long treatment cycles, slow onset of action, high demands on patient compliance, and significant relapse upon discontinuation of medication. Therefore, immunomodulatory biomaterials could represent a new therapeutic approach for postmenopausal osteoporosis. In the future, if biomaterials can be used to release or modulate local signaling in an orderly and controlled manner, thereby precisely upregulating M2 macrophage markers (CD206, CD163) and promoting the polarization of M1 to M2 macrophages. We could then combine drugs with biomaterials to provide both local and systemic treatment simultaneously, thereby exerting a more effective and long-lasting effect on postmenopausal osteoporosis. Therefore, the use of immune-modulating biomaterials targeting macrophages for the treatment of osteoporosis is highly worthy of further research. Key message: By elucidating the roles of macrophages and estrogen in bone homeostasis, the targeted regulation of macrophage polarization to the M2 phenotype and the maintenance of this M2 state are key to restoring the immune-bone microenvironment homeostasis in postmenopausal osteoporosis.

## 1 Introduction

Postmenopausal osteoporosis is a clinically common disease that mainly occurs in middle-aged and elderly women. The significant decrease in estrogen levels affects bone metabolism related cells, leading to a decrease in bone mass and an increase in bone fragility, and ultimately osteoporosis (1). Estrogen plays an important role in bone formation, it promotes osteogenic differentiation of mesenchymal stem cells (MSCs) and stimulates osteoblast maturation, and also inhibits osteoclast formation and induces osteoclast apoptosis, thus maintaining the density and strength of bone (2). Estrogen can also upregulate the expression of osteoprotegerin (OPG) (3), a decoy receptor for RANKL. OPG inhibits the binding of RANKL to RANK on osteoclasts, thereby suppressing osteoclast activation (4). So after menopause, this balance between bone formation and bone resorption is disrupted as estrogen levels decrease, the inhibitory effect of estrogen on osteoclasts is weakened, resulting in a relative enhancement of osteoclast activity and stimulate osteoclast formation, ultimately leading to gradual loss of bone mass (5).

Estrogen not only directly affects bone cells, but also indirectly affects bone cells by regulating various immune cells. These immune cells, such as T lymphocytes (6), B lymphocytes, and macrophages participate in regulating bone formation and bone resorption (7). When estrogen is deficient, these immune cells are inclined to secrete cytokines that accelerate bone resorption and hinder bone formation, leading to a decline in bone density and an increase in bone fragility.

The effect of macrophages on bone cells is two-sided, and according to its different polarization states, it can have completely different effects on bone formation and resorption. According to different chemokines, macrophages can be polarized into the pro-inflammatory M1 and the anti-inflammatory M2 (8–10),and the M2 has many subtypes. M1 macrophages promote bone resorption (11) and initiate the vascular outgrowth program through cytokines such as IL-1, IL-6, IL-12, IL-23,VEGF and TNF-α (2), while M2 macrophages promote bone formation and blood vessel formation (11) by secreting cytokines such as IL-2, IL-10, TGF-β (2). Therefore, macrophages play an important role in both bone formation and bone absorption.

Nowadays, the clinical treatment of osteoporosis is still based on medication to slow down the loss of bone mass. However, it faces problems such as long treatment period, slow onset of effect, and high demand for patient compliance. Therefore, exploring new therapeutic strategies through immune modulation has become a significant research direction, such as those currently being investigated: targeting RANKL, inhibiting inflammatory cytokines, probiotics that regulate the gut-bone axis (12), and mesenchymal stem cell therapy (13, 14). However, these methods present new challenges such as limited intervention strategies and significant rebound effects upon discontinuation. In recent years, research on immunomodulatory biomaterials derived from bio-tissue engineering has gradually gained momentum. Immunomodulatory biomaterials are a class of specific biomaterials, 1.It can regulate the immune response and affect the activation state and differentiation of immune cells by interacting with the immune system in organisms to promote tissue repair, inhibit inflammation, enhance the ability to fight infection or treat specific diseases; 2.It can be used as a drug delivery system (8), as a carrier to deliver immunomodulators, drugs or vaccines to improve efficacy and reduce side effects; 3.It can also provide a suitable microenvironment to promote cell adhesion, proliferation and differentiation by emulating the physical and chemical properties of the extracellular matrix. Macrophages exhibit high sensitivity to various properties of immunomodulatory biomaterials. By modifying specific characteristics of these biomaterials, the polarization state of macrophages can be precisely regulated, thereby achieving the goal of preventing and treating postmenopausal osteoporosis.

## 2 Purpose

To answer the role of macrophages in the pathogenesis of postmenopausal osteoporosis, and the changes in the immune system after menopause, and to propose new ideas for the treatment of postmenopausal osteoporosis with immune-modulating biomaterials, the aims of this review are 1: to summarize the role of macrophages in osteoblasts, osteoclasts and angiogenesis in different states of polarization.2: to explore the effect of estrogens on the immune cells.3: to summarize the effect of biomaterials on the state of macrophage polarization in terms of the Regulation.

### 2.1 Effect of macrophage polarization on osteoblasts

As an important part of the innate immune system, macrophages play an important role in the fight against inflammation and tissue damage in the body. Macrophages can phagocytic cell debris and various foreign bodies (15), secrete various inflammatory cytokines (16), present antigens and recruit other immune cells (17). Therefore, when the body has acute inflammatory response and tissue damage, the phenotype of macrophages will be polarized into the pro-inflammatory M1 type; Macrophages also play an important role in the inhibition of inflammation and tissue recovery after inflammation, and the macrophage phenotype changes from the pro-inflammatory M1 type to the anti-inflammatory M2 type (8). Most of the cytokines secreted by M1-type macrophages tend to promote bone resorption and inhibit bone formation, whereas those secreted by M2-type macrophages are more inclined to promote bone formation and inhibit bone resorption (2, 11, 16, 18). The states of M0, M1, and M2 form a dynamic continuum of polarization states that can transition into one another (19, 20).

The maintenance of bone homeostasis relies on multiple cell types, among which osteoblasts directly promote bone growth. By synthesizing and secreting various macromolecular proteins, primarily collagen, osteoblasts construct the organic framework of the bone matrix. This framework provides the essential structural template for the directed deposition and mineralization of calcium phosphate (21), ultimately forming bone tissue. Given the limited lifespan of osteoblasts, continuous replenishment by precursor cells is required. One such source is bone marrow mesenchymal stem cells, which are a type of stem cell possessing self-renewal capacity and multipotent differentiation potential, primarily found in bone marrow, they can become precursors to osteoblasts (22–24), participating in the remodeling and repair of bone tissue. Osteocytes play a crucial role in the maintenance and signaling of the skeleton. These cells interact with each other to collaboratively maintain the health and stability of the bones.

#### 2.1.1 Effects of M1 macrophages on osteoblasts

M1-type macrophages are also called pro-inflammatory macrophages, secrete a large number of pro-inflammatory cytokines (25), as well as reactive oxygen species, to promote the inflammatory response within the region in order to phagocytose cellular debris, foreign bodies, and pathogens. The main cytokines secreted by M1-type macrophages include, but are not limited to, IL-1β, IL-6, and TNF-α (8), which inhibit osteoclasts in two major ways, one is to inhibit osteoclastic The first is to inhibit osteoblast differentiation, i.e., to reduce the number of osteoblasts: IL-6 inhibits osteoblast differentiation by activating the JAK/STAT, SHP2/MEK2, and SHP2/AKT signaling pathways (26). It also upregulates TNF-α in osteoblasts to suppress Wnt/β-catenin signaling, thereby inhibiting osteoblast differentiation (27, 28); Higher concentrations of TNF-α inhibit the expression of IGF-I and RUNX 2 (29, 30), and thus inhibit osteoblast differentiation (18). Secondly, inhibition of osteoblast function: IL-1β was able to activate p38 MAPK to affect human osteoblast function and inhibit osteoblast migration. However, studies have also demonstrated that lower concentrations of TNF-α (0.01–1 ng/ml) can upregulate the expression of BMP-2, Runx2, Osterix, and osteocalcin through the NF-κB signaling pathway, thereby promoting osteogenic differentiation (31). Thus, TNF-α inhibits osteogenic differentiation under long-term normal or high concentrations, while it can transiently promote osteogenesis under short-term low concentrations.

#### 2.1.2 Effects of M2 macrophages on osteoblasts

The M2 phenotype of macrophages is mainly responsible for relieving inflammation and promoting tissue regeneration (25), so M2 macrophages are also known as anti-inflammatory macrophages. There are four subtypes of M2 macrophages, M2a, M2b, M2c, and M2d (32–35), which are able to secrete numerous cytokines, such asIL-4, IL-10, IL-12, TGF-β, IGF-1 (36), and PDGF and many other cytokines to control inflammation and promote osteoblast differentiation and recovery of peripheral blood vessels and tissues. M2a macrophages secrete IL-10, IGF-1 PDGF, TGF-β, etc. These cytokines mainly promote osteoblast differentiation, i.e., increase the number of osteoclasts. Physiological concentrations of IL-10 can cellularly promote the differentiation of human bone marrow mesenchymal stem cells to osteoblasts (37); IGF-1 stimulates the proliferation of osteoblasts through the MAPK and PI3K-Akt pathways (38); TGF-β can promote osteoblast proliferation, chemotaxis, and early differentiation through the TβRI/ALK5 signaling pathway in the early stages, thereby increasing the number of osteoblasts (39). Osteoprogenitor cells typically originate from bone mesenchymal stem cells, possessing strong osteogenic differentiation potential and serving as precursor cells capable of differentiating into osteoblasts (22–24). M2b macrophages can secrete IL-10, TNF-α, etc., of which IL-10, as indicated above, can cellularly promote the differentiation of human bone marrow mesenchymal stem cells to osteoblasts and thus increase the number of osteoblasts; M2c and M2d macrophages can also promote osteoblast differentiation through the aforementioned mechanisms, but they exert a more pronounced effect on angiogenesis (33).

### 2.2 Effect of macrophage polarization on osteoclasts

Osteoclasts are an important type of multinucleated cell in bone tissue, primarily responsible for bone resorption and remodeling. They originate from monocytes in the bone marrow and undergo a series of differentiation and fusion processes to ultimately form osteoclasts. Osteoclasts degrade bone matrix by secreting acidic substances and enzymes such as collagenase. While normal osteoclast function is essential for maintaining bone homeostasis, their excessive activity can lead to diseases like osteoporosis. The formation and activity of osteoclasts are regulated by various factors, particularly RANKL and OPG. During the formation of osteoclasts, the binding of RANK to RANKL is critical (40). RANK is the receptor activator of nuclear factor κB, expressed by osteoclast precursors and mature osteoclasts (41), while RANKL is the ligand for RANK, expressed by osteoblasts (42). Once RANKL binds to RANK, it activates the differentiation of osteoclast precursors, promoting their transformation into mature osteoclasts. Under the stimulation of RANKL, mature osteoclasts enhance their bone resorption capacity. Therefore, the RANKL/RANK pathway is significant for studying bone metabolism-related diseases, this may lead to the occurrence of osteoporosis.

#### 2.2.1 Effects of M1 macrophages on osteoclasts

As mentioned above: M1 macrophages are usually associated with the promotion of inflammation (43), mainly in the promotion of osteoclast production and activation, which affects osteoclasts through the secretion of inflammatory cytokines such as IL-1β, IL-6, IL-12, IL-23, TNF-α (8), and so on. These cytokines can promote osteoclast differentiation, i.e. increase the number of osteoclasts, e.g. IL-1βupregulates RANKL production (44, 45) and promotes RANK-RANKL binding, which in turn stimulates osteoclast formation; TNF-α promotes the induction of RANKL expression (46), and also stimulates osteoclast precursors to increase RANK expression (47), thereby increasing the binding of RANK to RANKL, which indirectly promotes the differentiation of osteoclast precursors into mature osteoclasts (48); and TNF-α can act directly on the surface receptors of osteoclast precursor cells and activate, in turn, NF-κB, p50/p52, c-Fos and nuclear factors activated NFATc 1 to induce osteoclast precursor cell differentiation (49, 50), *in vitro*, it promotes RANKL-induced osteoclast formation by activating NF-κB and PI3K/Akt signaling pathways (51). IL-6 also promotes osteoclasts by enhancing the expression of RANKL on the surface of osteoclasts (52) and by increasing the binding of RANKL to RANK formation; IL-23 promotes osteoclast differentiation by both upregulating RANK expression in bone marrow precursor cells and increasing RANKL expression in CD4(+) T cells (53), it also participates in T cell-mediated osteoclast differentiation (9, 54);In addition to increasing osteoclast differentiation, s Research indicates that IL-1β significantly increases during estrogen deficiency. It activates lyso-PAF acetyltransferase in osteoclasts, thereby enhancing PAF synthesis. PAF binds to its specific receptor on the osteoclast surface, activating downstream PI3K/Akt and ERK pathways, which can extend osteoclast survival (55, 56). However, M1 macrophages also secrete cytokines such as IL-27 that inhibit osteoclasts. IL-27 can reduce osteoclast numbers by affecting their maturation through suppressing Th17 cells and RANKL expression (57).

#### 2.2.2 Effects of M2 macrophages on osteoclasts

**Figure 1:**
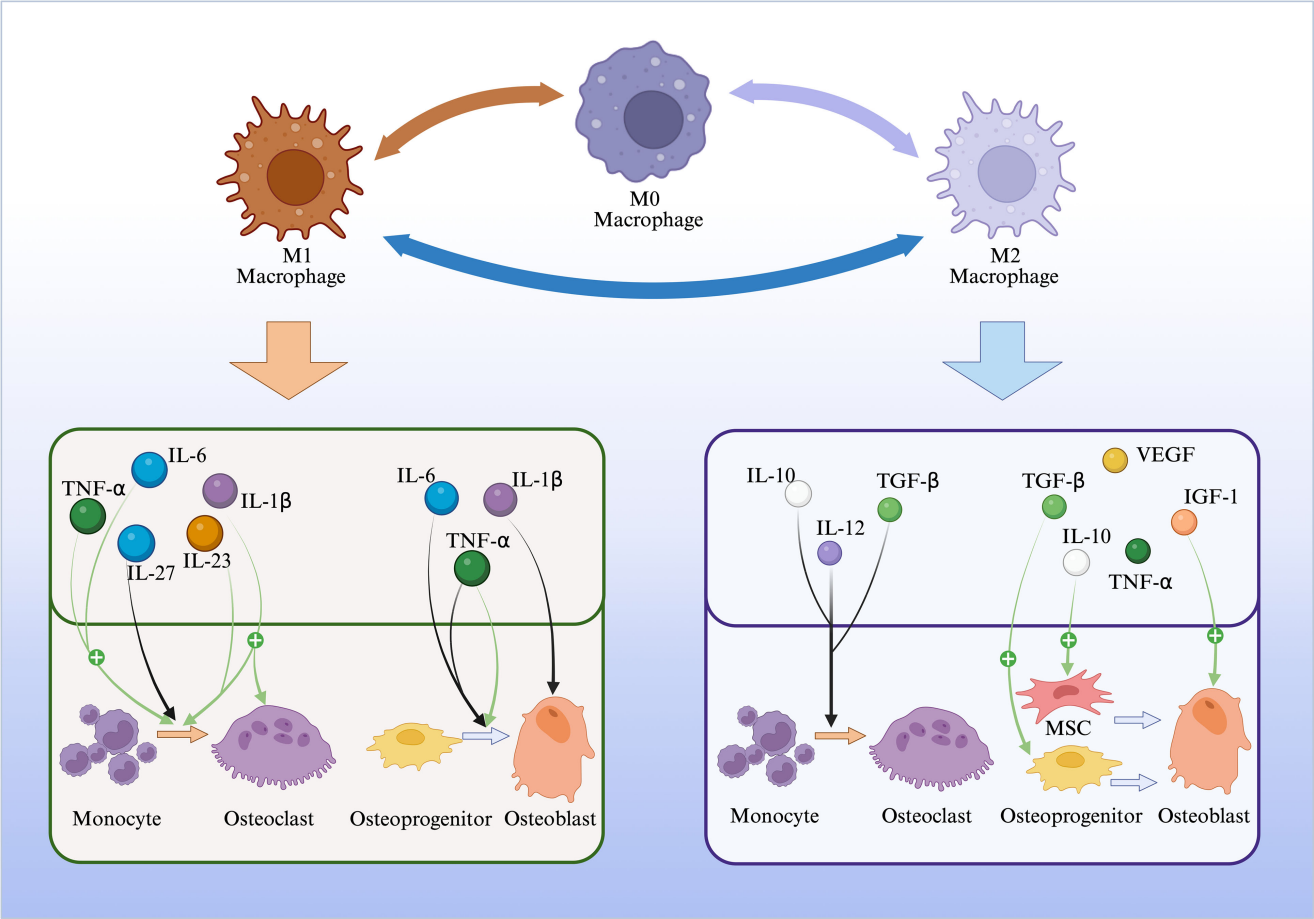
The effects of two macrophage polarization states on osteoblasts and osteoclasts.

M2-type macrophages and the cytokines they secrete are mainly responsible for removing inflammation and repairing tissues (43), and play an inhibitory role in osteoclasts. The cytokines secreted by M2-type macrophages mainly include IL-4, IL-10, TGF-βsecreted by M2a, etc., and these cytokines inhibit osteoclasts differentiation, reduce the number of osteoclasts; IL-4 inhibits osteoclast generation by downregulating RANKL expression in osteoblasts (58), thereby reducing RANK-RANKL binding, and also suppresses osteoclast differentiation by inhibiting mitogen-activated protein kinase(MAPK) (59). IL-10 synergistically inhibits osteoclastogenesis through multiple pathways: at the signal initiation stage, It can both upregulate OPG expression and suppress RANK expression (60), thereby doubly blocking the RANKL-RANK binding pathway; At the intracellular signaling level, it suppresses key osteoclast differentiation programs by inhibiting c-Fos/c-Jun and sNFATc1 activity (61), while also leveraging TREM-2’s transcriptional repression (47). Additionally, TGF-β primarily inhibits osteoclast differentiation from a systemic balance perspective by downregulating the RANKL/OPG ratio (**Figure 1**).

### 2.3 Effects of macrophages on blood vessels

Macrophages are closely associated with maintaining vascular patency, initiating vascular sprouting, and promoting anastomoses in new blood vessels. Research indicates that within the dermis layer of the skin, macrophages can maintain capillary patency and delay vascular network degeneration through CX3CR1-mediated directional recruitment and RAC1-dependent phagocytic clearance mechanisms, thereby preventing a decline in capillary perfusion function (62). Similarly, within bone tissue, macrophages mediate the formation of new blood vessels, as well as the expansion and anastomosis of these neovessels. This facilitates the development of a rich vascular network within bone tissue, delivering essential nutrients and oxygen to support the metabolic activities of various cells within the bone matrix.

Vessels can also influence osteoblasts and osteoclasts, vascular tissue not only stimulates osteoblast migration and differentiation by secreting various factors that bind to receptors such as VEGFR on the osteoblast surface (63), but also inhibits osteoblast apoptosis (64); It also promotes the recruitment and migration of osteoclasts, and can bind to KDR/Flk-1 or Flt-1 receptors on osteoclast membranes to directly enhance osteoclast function and survival (65). Furthermore, the synergistic interaction between vascular endothelial cells and osteoclasts collectively contributes to the formation of the bone marrow cavity (66). Therefore, both the processes of bone formation and bone remodeling depend on the support of a fully functional vascular network.

Research indicates that activated macrophages inoculated in guinea pig corneas induced intense vascular proliferation (15). A large number of macrophages can also be observed in tumor tissue, so an increase in the number of macrophages is associated with increased angiogenesis (67). If macrophages are reduced or even depleted, angiogenesis will be significantly decreased (68–70). For example, in tumor-bearing mice, macrophage depletion inhibits tumor blood vessel formation (71). All of the above experiments demonstrate that macrophages have an important role in blood vessel formation, thus suggesting that macrophages are an important source of pro-angiogenic signals. Different polarization states of macrophages exert distinct effects on blood vessels. M1 macrophages serve as initiators of vascular sprouting, responsible for triggering angiogenesis. They highly express factors such as VEGF, FGF2, IL-8, TNF-α, and IL-1β to promote endothelial cell sprouting. M2a cells highly express PDGF, which recruits pericytes (72) and mesenchymal stem cells (73) to the site of neovascularization. This effectively stabilizes the newly formed vascular system and promotes its anastomosis. M2c macrophages secrete elevated MMP9 to support vascular sprouting and remodeling (74).

Macrophages can promote the activation, proliferation and survival (15, 75) of endothelial cells by secreting various growth factors and inflammatory cytokines, thereby promoting the formation of new blood vessels (76). thereby promoting the formation of new blood vessels and supporting their stability and longevity. Macrophages can also interact with the anastomotic ends of newly formed blood vessels, promoting the anastomosis between these vessels (15). In aortic ring experiments in mice, macrophages promote growth factor production and modulate the extracellular matrix after establishing physical contact with endothelial cells of the neovascularization, thereby creating a more suitable environment for vascular anastomosis between the new blood vessels (77). In the bone tissue of osteoporosis patients, the number of blood vessels is typically reduced, and their structure may become irregular (78). This can lead to decreased local blood flow, impairing the supply of oxygen and nutrients to bone tissue (79). Consequently, it further accelerates the progression of osteoporosis, intensifies inflammatory responses, and disrupts the transport of osteoblast precursors and osteoclast precursors, thereby affecting the bone remodeling process.

**Figure 2:**
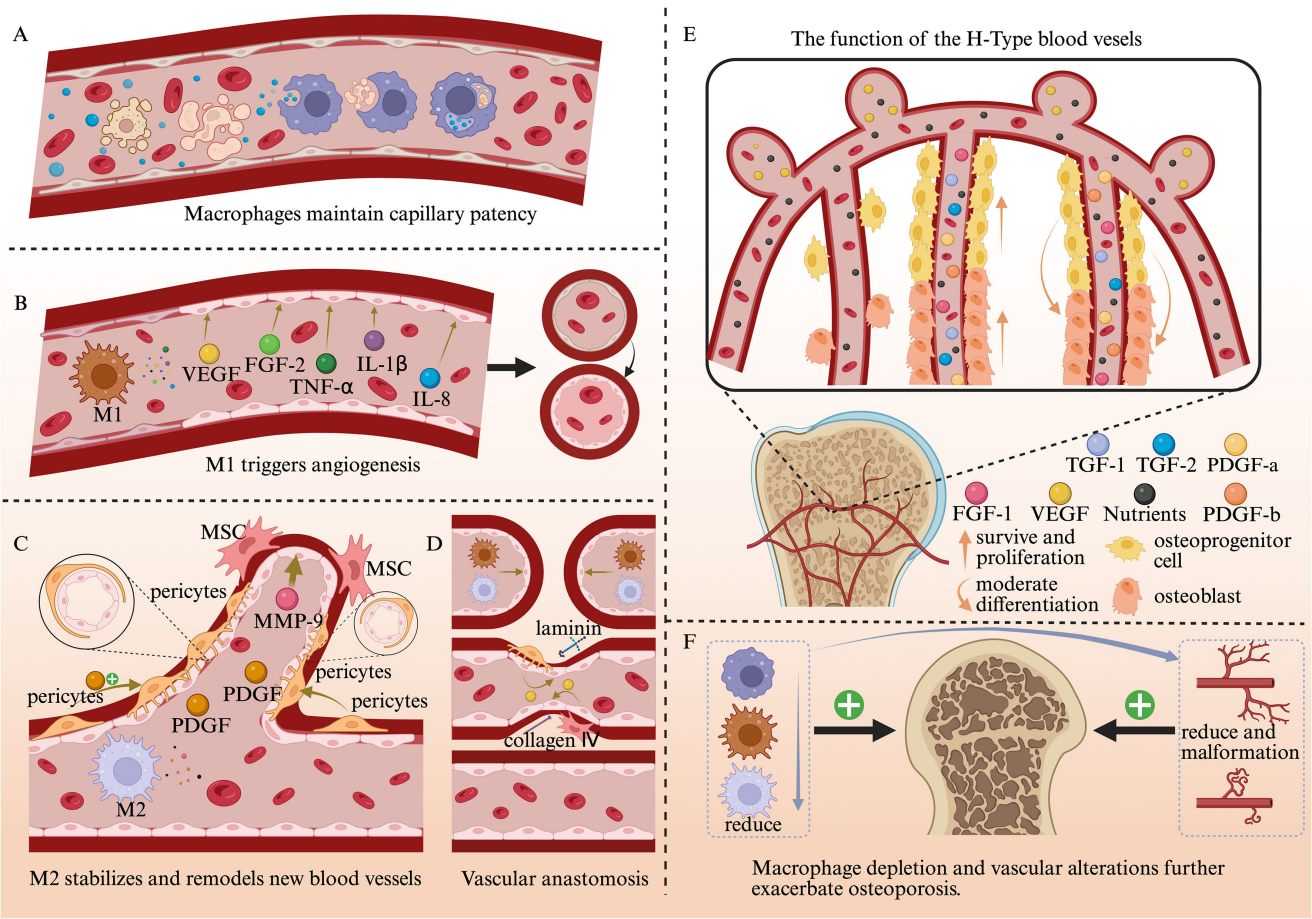
The multifaceted regulatory roles of macrophages in maintaining vascular patency and the remodeling of newly formed blood vessels, as well as their crucial role in bone formation **(A)** Macrophages clear foreign substances and necrotic/senescent cells, maintaining vascular patency. **(B)** M1 macrophages act on vascular endothelial cells to initiate vascular sprouting. **(C)** M2 Macrophages stabilize and remodel new blood vessels. **(D)** Macrophages promote neovascular anastomosis. **(E)** The Positive Effect of H-Shaped Blood Vessels on Bone Formation. **(F)** Macrophage depletion and vascular alterations further exacerbate osteoporosis.

Blood vessels within bone tissue not only deliver nutrients but also participate in regulating osteogenesis and bone regeneration. Vessels located at the metaphysis and arranged in a columnar pattern along the periosteum, with terminal tubular arches and blind-ended globular protrusions, are called H-type vessels. These vessels highly express growth factors such as PDGFa/b, TGF1/3, and FGF1, causing osteoprogenitor cells and osteoblasts to cluster densely around them. This supports osteoprogenitor and osteoblast survival and proliferation (78). In H-type blood vessels within bone tissue, Notch signaling not only promotes endothelial cell proliferation and vascular growth but also regulates the appropriate differentiation of early bone progenitor cells into osteoblasts by expressing the BMP antagonist Noggin. This maintains the bone progenitor cell pool, ensuring stable and sustained bone formation (80). The latest research reveals that the generation of H-type vessels within bones and their close association with bone metabolism are key to preventing and treating postmenopausal osteoporosis (81) (**Figure 2**).

### 2.4 Effects of estrogen on the immune system and blood vessels

Estrogen, as a crucial immunoregulatory hormone, exerts its effects across multiple levels of the immune system: Estrogen exerts broad effects on diverse immune cells, while also participating in regulating macrophage polarization (82). Furthermore, it directly modulates bone metabolism-related signaling pathways and indirectly intervenes in disease processes such as osteoporosis by suppressing chronic inflammation. Consequently, estrogen plays a vital role in maintaining the equilibrium between immune function and bone metabolism.

#### 2.4.1 Effects of estrogen on the immune system

Estrogen enhances T-cell (6) and B-cell-mediated (83) adaptive immunity (84), increases antibody production (85), and simultaneously suppresses excessive inflammation triggered by neutrophils and other cells (86), thereby synergistically maintaining the body’s immune state. Specifically, regarding macrophages, estrogen tends to protect M2 macrophages (87). In the presence of estrogen, M2 macrophages maintain their role in promoting osteoblast differentiation while inhibiting osteoclast differentiation. Once estrogen withdrawal occurs, M2 macrophages cease to sustain their original function and instead differentiate into functional osteoclasts (88). To study the effects of estrogen on macrophages, Ce Dou’s team removed the ovaries from mice to establish a mouse model of osteoporosis and found an increased M1/M2 macrophage phenotype ratio in the bone marrow of ovariectomised C57 BL/6 mice. Bone marrow macrophages were then extracted from mouse femurs and polarised into M1 and M2 types, and then exposed to RANKL stimulation, and it was found that M2-type macrophages could differentiate into functional osteoclasts in the absence of estrogenic protection, whereas M1-type macrophages could not (88). OVX mice were then pretreated with 17β-estradiol (E2), E2 protects M2 macrophages from RANKL stimulation by binding to estrogen receptor-α and downstream inhibition of NF-κB p65 nuclear translocation (9), which prevents the differentiation of M2-type macrophages to osteoclasts. Postmenopausal women have a significantly impaired response to M2-type macrophage-associated stimulants (IL-4, IL-13) and no significant change in response to M1-type macrophage-associated stimulants (LPS, IFN-γ), which results in a significantly higher ratio of macrophage phenotypes M1/M2 (89), which will be more pronounced for M1-type macrophages than for M2-type macrophages (84). Estradiol pretreatment of human macrophages *in vitro* was found to inhibit the NF-κB signaling pathway and lipopolysaccharide (LPS)-induced TNF-α production (84, 89), and it has also been experimentally demonstrated that estrogen enhances the production of IκB-α, an endogenous NF-κB inhibitor (90), both of which demonstrated that macrophages in an estrogenic milieu reduce TNF-α secretion. Reduced secretion of TNF-α is effective in delaying the onset and progression of osteoporosis. Therefore, estrogen regulates macrophages by promoting M2 polarization and suppressing M1 polarization (87, 91), thereby reducing inflammatory responses and facilitating tissue repair.

RANK binding to RANKL has been identified as an effective stimulus for osteoclasts (92), increasing osteoclast differentiation and maturation and decreasing osteoclast apoptosis. RANKL is essential for osteoclast formation, but a certain amount of m-CSF is also required to activate osteoclasts (9, 93). Whereas at the same time osteoblast lineage cells are able to secrete osteoprotegerin (OPG) (47) to reduce the binding of RANK to RANKL (94). In this process, estrogen reduces the production of RANK (95) and macrophage colony-stimulating factor (M-CSF) on the one hand, to reduces the formation of osteoclasts in this pathway. On the other hand, estrogen increases the production of the OPG (94, 96), which reduces the formation of osteoclasts to achieve bone protection (3). Estrogen can also directly reduce the number of osteoclasts by inducing their apoptosis (97). This is a direct effect on the process of bone formation and resorption. In postmenopausal osteoporosis, decreased estrogen levels lead to increased RANKL (98), which then increases bone resorption.

The indirect effect of estrogen on the process of bone resorption and formation is through the regulation of cytokine secretion, i.e. by acting on monocytes/macrophages and reducing their formation of IL-1, IL-6 and TNF (84). It can be seen that estrogen can effectively inhibit the polarization of M1-type macrophages (86) and reduce the release of inflammatory factors that can promote bone resorption, thereby reducing bone resorption. Not only that, but estrogen has a protective effect on M2-type macrophages, which in turn contributes to bone formation. Surgery-induced estrogen deficiency or estrogen deficiency due to natural menopause both lead to excessive production of IL-1, IL-6, TNF-α by osteoblasts. Thus when postmenopausal women are estrogen deficient, TNF-α significantly promotes the expression of RANKL, M-CSF to increase osteoclast differentiation.IL-6 can promote osteoclast differentiation by increasing the expression of RANKL to increase the number of osteoclasts, and also enhances their osteoclast function by increasing osteoclast activity. Estrogen, on the other hand, plays a role in up-regulating the expression of TGF-β, thus acting on osteoclasts, decreasing their activity, increasing their apoptosis, and thus slowing down the process of bone resorption.

#### 2.4.2 Effects of estrogen on blood vessels

Bones need sufficient blood to supply the necessary nutrients, so the formation of blood vessels is essential for nutrients to reach the bones. The formation of new blood vessels promotes bone remodeling, reduces chronic inflammation and provides nutrients and hormones, of which estrogen not only promotes bone formation and inhibits bone resorption as mentioned above, but also has a positive impact on the formation of blood vessels (99), which provide nutrients to the bones and effectively slows down the onset of osteoporosis. This provides nutrients to the bones and effectively slows down the onset of osteoporosis. Estrogen can directly stimulate the synthesis and release of nitric oxide (NO) in the vascular wall (100), which is important for vascular endothelial growth factor (VEGF)-induced angiogenesis, as well as stimulating VEGF production in vascular tissues. Estradiol promotes endothelial cell proliferation *in vivo* and *in vitro*, and has significant vasculoprotective and anti-inflammatory effects (101), whereas high doses of estradiol also promote reendothelialization in castrated female rats. Morales, David E’s team demonstrated in *in vitro* experiments on human umbilical vein endothelial cells that 17β-estradiol is able to promote angiogenesis by facilitating cell attachment, migration, proliferation and differentiation (102). In addition, estradiol is capable of inducing collateral vessel formation (84). As mentioned above, estrogen protects M2-type macrophages, M2 macrophages exert a significant effect on angiogenesis (103, 104), and the formation of new blood vessels delivers not only nutrients and hormones, but also bone morphogenetic proteins(BMP), insulin-like growth factor(IGF) and other cytokines, which have a direct impact on the activity of osteoblasts and osteoclasts.

**Figure 3:**
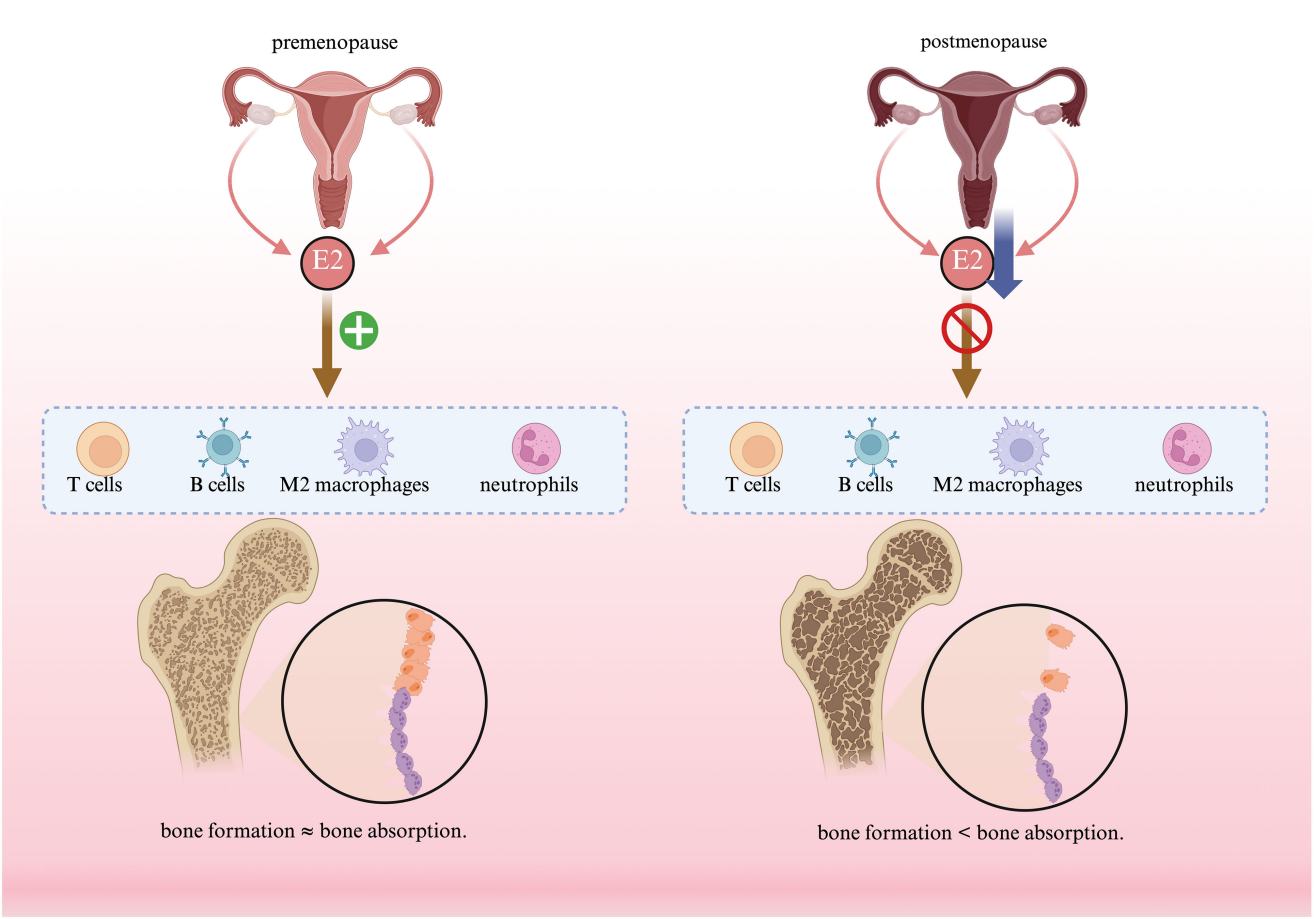
The effects of estrogen on immune cells and bone estrogen interacts with various immune cells to help maintain bone resorption and bone formation.

It is thus clear that, in addition to the previous pathogenesis, estrogen is also able to cause osteoporosis by affecting immune cells. Among the various immune cells influenced by estrogen, macrophages play an important role in regulating the bone remodeling process. Therefore, if the polarization state and biological behavior of macrophages can be regulated, this could provide a new therapeutic direction and strategy for the treatment of postmenopausal osteoporosis, combining with emerging biomaterials at this stage, to form immunomodulatory biomaterials. By adjusting the properties of these biomaterials or delivering estradiol through them, macrophage polarization can be induced, modulate cytokine secretion, and inhibit macrophage-to-osteoclast conversion. This approach could pioneer a novel therapeutic strategy for postmenopausal osteoporosis (**Figure 3**).

## 3 Modulation of macrophages by biomaterial

In the treatment of bone-immune disorders, biomaterials repair bones by providing structural support and guiding regeneration. Those with immune-modulating capabilities demonstrate even greater potential. By actively modulating immune responses, these materials can target key mechanisms in disease progression, holding significant promise for the prevention and treatment of diseases (14). When biomaterials are implanted into the body they cause a certain foreign body response (8, 105), and macrophages are the first immune cells to interact with biomaterials and are an important mediator of the foreign body response that constitutes the host’s response to biomaterials. Following the implantation of biomaterials, macrophages polarize into the M1 phenotype and secrete cytokines, coordinating the inflammatory response at the implant site, it is essential to promptly shift the macrophage phenotype from M1 to M2—a phenotype that promotes tissue remodeling and anti-inflammation. This shift helps regulate bone homeostasis and halt ongoing bone resorption. This type of immunomodulatory biomaterial, which can regulate the ratio of M1 to M2 macrophages, plays an important role in bone repair and regeneration.

### 3.1 Pathways of macrophage polarization

Macrophage polarization involves a complex network of molecular regulatory mechanisms. M1 polarization is primarily mediated by classical signaling pathways such as NF-κB/STAT1, TLR/IRF, and JAK-STAT1, as well as signaling axes downstream of GM-CSF, including JAK2-STAT5, PI3K-AKT, and Ras-MAPK; In contrast, M2 polarization is primarily regulated by the synergistic action of pathways such as NF-κB/STAT6, JAK1-STAT6, CSF-1/IL-34, PPARγ/PPARδ, KLF2/KL4, and IL-10/STAT3 (19, 106).

### 3.2 The physical properties of biomaterials modulate macrophages

The surface morphology of biomaterials plays a crucial role in regulating macrophage polarization states, including factors such as surface roughness, hardness, presence of pores, pore size, and pore density. The effect of biomaterial surface roughness on macrophage polarization varies across experiments. *In vitro* culture of J774 A.1 macrophages revealed that increased surface roughness significantly elevated BMP-2 expression (107), indicating a tendency toward M2-type polarization. This finding holds significant implications for promoting osteogenic differentiation on biomaterial surfaces. Primary 264.7 macrophages cultured on sandblasted and acid-etched titanium surfaces can be activated to an M2-like phenotype (108). However, other studies indicate that increasing roughness may also promote M1 polarization. In an experiment with primary 264.7 macrophages cultured *in vitro*, as the roughness of the titanium surface increased from 100 to 400 nm, the cells progressively shifted toward an M1 phenotype (109). *In vitro* culture of THP-1-derived macrophages revealed that as the surface roughness of mineralized gelatin increased from 0.92 µm to 12 µm, macrophages polarized toward the M1 phenotype on rougher surfaces (110). Therefore, the influence of biomaterial surface roughness on macrophage polarization is not unidirectional; its effects are highly dependent on the combined actions of specific roughness scales, morphological features, and the biological environment.

Within collagen-based matrices systems, higher matrix stiffness typically promotes M2 polarization of macrophages (8). Studies indicate that, culturing human macrophages on collagen-based substrates of varying stiffness revealed that increased substrate stiffness promotes greater polarization of macrophages toward the M2 phenotype (111). The incorporation of bentonite XLS into alginate-based polyacrylamide hydrogels enhances their rigidity, thereby promoting the M1-to-M2 macrophage polarization in these cultured cells (112).

The pore size and quantity on the surface of biomaterials also influence macrophage polarization. Biomaterials with larger pore sizes, such as those featuring 30–40 micrometer apertures (8), can significantly promote the anti-inflammatory M2 polarization of macrophages (113), increasing the proportion of M2 macrophages, while smaller pore sizes tend to lead to polarization towards the M1 type. This is because porous structures with appropriately sized pores can provide a larger surface area and space, facilitating cell attachment, migration, and interaction, thereby affecting cellular signaling and gene expression. The pores on the surface of biomaterials also influence the polarization of macrophages.

### 3.3 The chemical properties of biomaterials modulate macrophages

The chemical properties of biomaterials are also important for macrophage regulation. The charge carried on the surface of biomaterials, i.e. the surface anionic and cationic state, plays an important role in the regulation of macrophage activity. Macrophages have negatively charged cell membrane surfaces, and if biomaterials can regulate the surface charge of macrophages, they can alter their adsorption of proteins, thereby affecting their biological behavior (114). Tests were performed on macrophages, showing that when the surface of the material is positively charged, macrophages can be polarized to the M1 type through the Toll-like receptor 4 (TLR-4) pathway. Experiments have demonstrated that cationic polymers such as polyethyleneimine (32) and cationic gelatin, can reverse the polarization of tumor-associated macrophages (TAMs), shifting them from the M2 phenotype to the M1 phenotype (114). Cationic polymers significantly enhance IL-12 expression and suppress IL-10 expression in TAMs and macrophages both *in vitro* and *in vivo*. Surface charge also influences early inflammatory responses at biomaterial implantation sites (115). Materials with cationic surfaces facilitate more efficient and rapid macrophage recruitment compared to anionic or neutral surfaces (116).

Surface chirality of biomaterials can also play a significant role in macrophage regulation (114). The two enantiomers of N-Isobutyryl-L(D)-cysteine (NIBC), L-NIBC and D-NIBC, were used to modify the gold-plated surface as a model system to study the effect on macrophages, and the results showed that the number of macrophage adhesion was large on the surface of L-NIBC, and the majority of these macrophages would exhibit stretching, deformation, protruding pseudopods, and clustering together, and these manifestations indicate macrophage activation to the pro-inflammatory M1 type. On the other hand, the number of macrophages adhering to the surface of D-NIBC was much less than that adhering to the surface of L-NIBC, and the macrophages on the surface of D-NIBC were scattered round macrophages, i.e., anti-inflammatory M2-type macrophages (117). Thus, on the L-surface (left-handed) macrophages adhere in large numbers and are polarised to the M1 type, whereas on the D-surface (right-handed) macrophages adhere in small numbers and are polarised to the M2 type.

**Figure 4:**
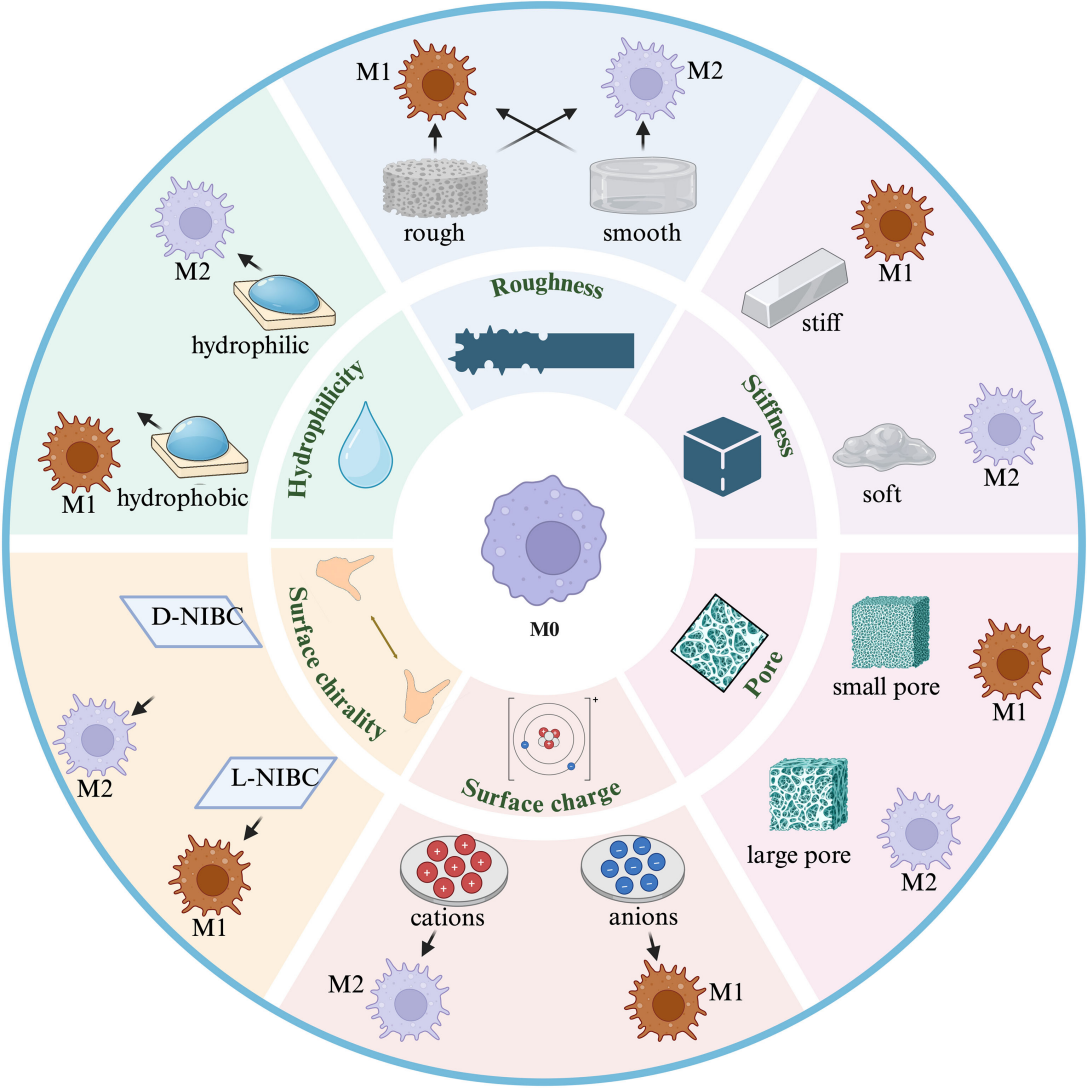
Regulation of macrophage polarization states by the physicochemical properties of biomaterials.

Hydrophilic functional groups are key determinants of the hydrophilicity of biomaterial surfaces, and surface hydrophilicity significantly regulates macrophage polarization. Therefore, precise control of surface functional groups can effectively modulate macrophage polarization. Cultivating macrophages on hydrophilic material surfaces revealed that it inhibits macrophage adhesion and spreading (118), reduces pro-inflammatory cytokine release, increases anti-inflammatory cytokine release, and induces M2 polarization (119, 120), thereby enhancing anti-inflammatory and reparative functions. For example, hydrophilic titanium sheets prepared in a 10% H_2_O_2_ solution promoted the growth of mouse macrophages, with detectable increases in IL-10 and decreases in TNF-α, indicating macrophage polarization toward the M2 phenotype (8). The hydrophilic oxide layer on the titanium surface can also prolong the M2 phenotype of macrophages (121). In contrast, hydrophobic surfaces tend to induce M1 type polarization (122), increasing the inflammatory response. Therefore, by regulating the surface hydrophilicity of biomaterials, it is possible to effectively influence the polarization state of macrophages, thereby improving the biocompatibility and functional performance of the materials (**Figure 4**).

### 3.4 The regulatory effects of biomaterial delivery on macrophages

Biomaterials can deliver drugs, enzymes, or bioactive elements through physical adsorption (porous scaffolds, gelatin sponges, hydrogels, nanoparticles), chemical bonding (forming chemical bonds), and surface coatings with degradation or release properties. Their delivery function can actively modulate the immune microenvironment at the implantation site, inducing macrophage polarization toward the reparative M2 phenotype, thereby creating favorable conditions for bone regeneration (123).

Physical delivery strategies can precisely regulate macrophage polarization. Studies confirm that using decellularized bone scaffolds to transiently release IFN-γ and IL-4 can modulate macrophage polarization toward M1 and M2 types (124). As a multifunctional delivery platform, hydrogels can not only load lysozyme to relieve inflammation (125) but also deliver factors such as IL-4 and E2 to directly induce M2 macrophage polarization, thereby efficiently promoting osteogenesis (126).

Inorganic bioactive coatings represent an effective approach to implement this strategy. For instance, β-TCP coatings guide macrophage polarization toward the M2 phenotype through their degradation products while upregulating bone morphogenetic protein 2 (BMP2) expression. This creates a microenvironment that combines anti-inflammatory and pro-osteogenic functions, significantly enhancing the osteogenic differentiation of bone marrow mesenchymal stem cells (BMSCs) (127). Similarly, degradation products from calcium borosilicate (Ca_11_Si_4_BO_22_) coatings suppress TLR signaling pathways, driving macrophage transition from M1 to M2 phenotypes and positively regulating BMSC osteogenesis (128). Delivering specific bioactive substances to targeted pathological environments demonstrates potential for precise immune modulation. In orthopedic metal implants, wear particles induce chronic inflammation dominated by M1 polarization, leading to periprosthetic bone resorption and loosening (129). Addressing this, Yang’s team successfully reversed M1 to M2 macrophage polarization by loading lithium chloride (LiCl) onto material surfaces. This not only improved the inflammatory environment but also significantly enhanced BMSC osteogenic capacity both *in vitro* and *in vivo* (130).

### 3.5 Osteogenic activity of biomaterials in the osteoporotic microenvironment

In the treatment of osteoporosis, biomaterials play a crucial role. Currently, there are various commercial biomaterials available on the market, including metallic materials such as titanium, which can provide necessary mechanical support and enhance factors like TGF-β and OPG through surface hydrophilicity and roughness, thereby increasing osteoblast expression and promoting bone tissue integration (131). Additionally, biocompatible gel materials like polylactic acid (PLLA) and polyvinyl alcohol (PVA) are widely used in bone repair due to their excellent biodegradability, ability to interact with cells, and drug delivery capabilities, effectively stimulating the proliferation and differentiation of relevant cells (114). Meanwhile, inorganic biomaterials such as bioactive glass and hydroxyapatite have shown outstanding performance in improving the healing of bone defects, as they can form chemical bonds with surrounding bone tissue and promote bone regeneration. The comprehensive application of these biomaterials not only significantly enhances the quality of life for patients with osteoporosis but also opens new research directions for future bone repair strategies.

## 4 Immunotherapy for osteoporosis

**Figure 5:**
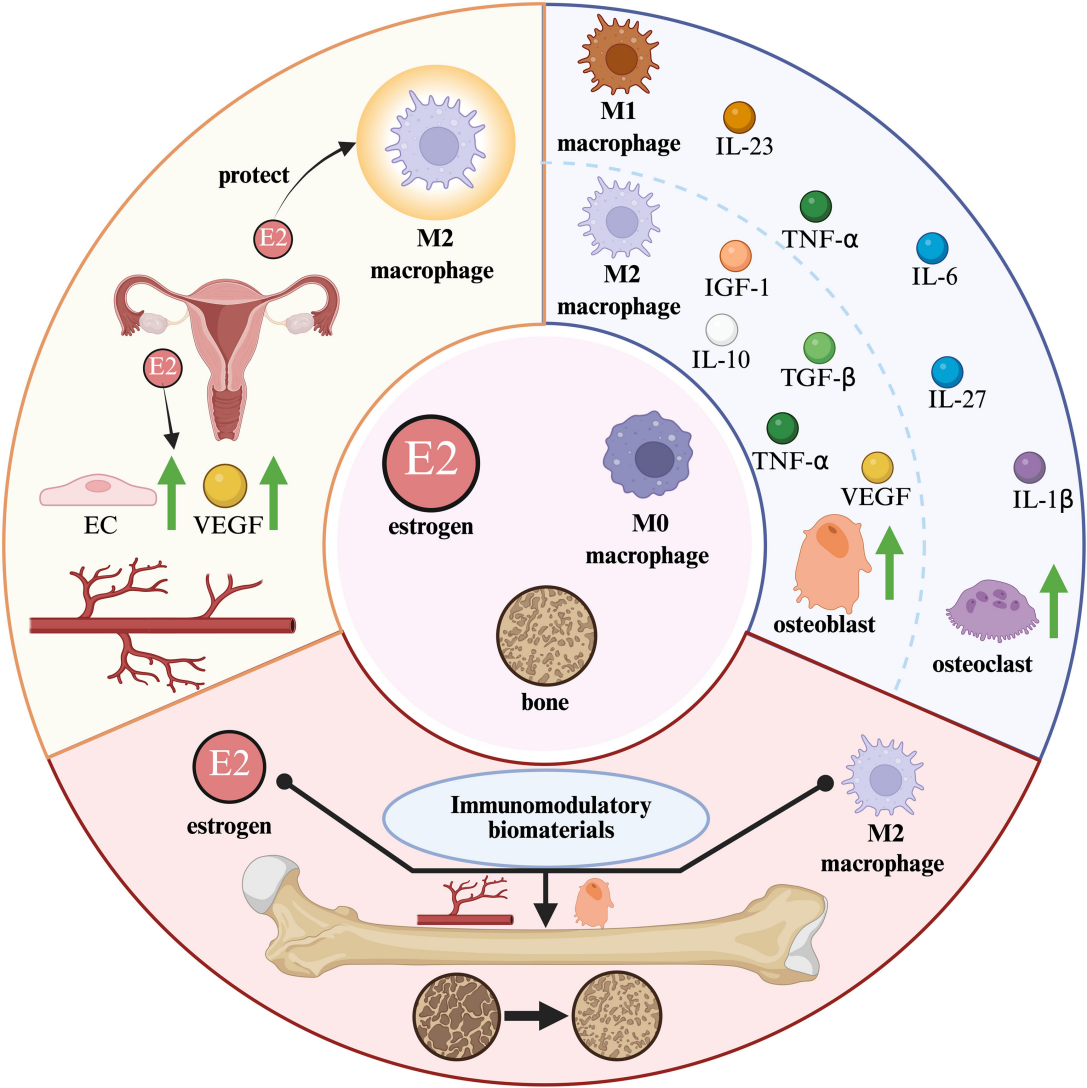
Summarizing the effects of estrogen and differences in macrophage polarization to design immunomodulatory biomaterials **(A)** Estrogen exerts a protective effect on M2 macrophages and promotes angiogenesis. **(B)** M1 macrophages primarily promote osteoclast differentiation and function, while M2 macrophages primarily promote osteobålast differentiation and function. (C) Immunomodulatory biomaterials can prevent and treat postmenopausal osteoporosis by regulating macrophage polarization and carrying estrogen.

Treatment strategies for osteoporosis are continually expanding, with immunotherapy gaining significant attention due to its potential in regulating bone remodeling. This approach targets specific immune cells or signaling proteins to intervene in key pathways such as RANK-RANKL-OPG, thereby restoring skeletal metabolic balance. It has emerged as a current research hotspot. Denosumab, a commonly used monoclonal antibody that targets RANKL (132), inhibits RANKL activity to slow down the destruction of osteoclasts, thereby reducing the rate of bone resorption and effectively treating osteoporosis (133). This medication has been widely applied in the treatment of osteoporosis, significantly lowering the risk of fractures. Additionally, Piezo1 is a mechanosensitive ion channel that prevents osteoporosis by inhibiting bone marrow adipogenesis and promoting osteogenic differentiation through suppression of the Ccl2-Lcn2 circuit in bone marrow mesenchymal stem cells (BMMSCs). It also serves as a key mediator of exercise’s beneficial effects on bone health (134). Therefore, future development of novel immunomodulatory drugs targeting Piezo1, such as Piezo1 agonists, holds promise for establishing a new paradigm in immunotherapy for this disease. In summary, biomaterials demonstrate promising therapeutic effects for localized osteoporosis. Those with immunomodulatory functions show even greater efficacy in treating systemic conditions like postmenopausal osteoporosis. By comprehensively reducing chronic inflammation, improving bone metabolism, and enhancing bone density, these materials hold significant promise in osteoporosis treatment, offering patients more effective therapeutic options (**Figure 5**).

## 5 Conclusion

In investigating the complex etiology and prevention strategies for postmenopausal osteoporosis, immune-skeletal interactions between macrophages and the bone microenvironment have garnered increasing attention. As key cells of the innate immune system, macrophages participate in bone homeostasis regulation through their phenotypic polarization: pro-inflammatory M1 macrophages secrete inflammatory cytokines that exacerbate bone resorption, while anti-inflammatory M2 macrophages support bone formation by releasing pro-osteogenic factors. Under estrogen-deficient conditions, the bone marrow microenvironment tends toward M1 polarization, disrupting bone remodeling equilibrium and accelerating bone loss. Therefore, elucidating the regulatory mechanisms of macrophages in bone metabolism not only provides new insights into the pathogenesis of postmenopausal osteoporosis but also lays a theoretical foundation for developing biomaterial therapeutic strategies centered on immune regulation. Future research could focus on designing immunomodulatory biomaterials or immunotherapeutic agents capable of precise spatiotemporal delivery. By selectively modulating macrophages to induce and maintain their M2 polarization, these agents could reshape the local microenvironment to promote bone formation, thereby opening new therapeutic avenues for the repair and improvement of osteoporosis.
